# Supplementary material for: The Possible Role of Prescribing Medications, Including Central Nervous System Drugs, in Contributing to Male-Factor Infertility (MFI): Assessment of the Food and Drug Administration (FDA) Pharmacovigilance Database
Source: Brain Sci. 2023 Nov 29;13(12):1652. doi: 10.3390/brainsci13121652 (PMC10741514; doi:10.3390/brainsci13121652)
Supplement: Supplementary file 1 [file brainsci-13-01652-s001.zip › brainsci-2659132-supplementary.pdf]

Table S1.

**Drug with a significant PRR**	**Number of individual cases associated with MFI in the database (n) Primary analysis**	**Number of individual cases associated with MFI in the database (n) Secondary analysis**	**Crude PRR (CI95%)**	**Adjusted PRR (CI95%)**
Finasteride	86	72	**16.04 (12.67 - 20.3)**	**12.94 (12.62, 13.27)**
Testosterone	33	31	**3.03 (2.12 - 4.32)**	**5.63 (5.54, 5.72)**
Valproic Acid	32	23	**1.72 (1.20 - 2.47)**	**3.53 (3.46, 3.61)**
Diethylstilbestrol	20	20	**14.3 (9.13 - 22.37)**	**17.98 (17.04, 18.95)**
Verapamil	14	12	**1.83 (1.07 - 3.12)**	0.79 (0.75, 0.82)
Nifedipine	12	10	**1.85 (1.04 - 3.28)**	0.95 (0.91, 0.98)
Mechlorethamine	17	14	**58.71 (36.30 - 94.94)**	0.57 (0.49, 0.66)
Lovastatin	13	9	**2.51 (1.44 - 4.36)**	**1.08 (1.04, 1.12)**

Keys: PRR = Proportional Reporting Ratio. CI95%= 95% Confidence Interval. MFI=male factor infertility. Adjusted PRR for concomitant products.
